# Rabies Postexposure Prophylaxis for Travelers Injured by Nonhuman Primates, Marseille, France, 2001–2014

**DOI:** 10.3201/eid2108.150346

**Published:** 2015-08

**Authors:** Agathe Blaise, Philippe Parola, Philippe Brouqui, Philippe Gautret

**Affiliations:** Institut Hospitalo-Universitaire Méditerranée Infection, Marseille, France (A. Blaise, P. Parola, P. Brouqui, P. Gautret);; Aix Marseille Université, Unité de Recherche sur les Maladies Infectieuses et Tropicales Emergentes, Marseille (P. Parola, P. Brouqui, P. Gautret)

**Keywords:** Rabies, nonhuman primates, rabies postexposure prophylaxis, viruses, France

## Abstract

Most exposures of residents of Marseille to nonhuman primates occurred among unvaccinated adult travelers to Southeast Asia within the first 10 days of their arrival at 2 major tourist locations in Thailand and 1 in Indonesia. A small proportion of travelers received rabies immunoglobulin in the country of exposure.

Rabies is estimated to cause >60,000 human deaths worldwide annually on the basis of a probability decision-tree approach; primarily resulting from dog bites, the disease is of public health concern in most countries in Asia and Africa (*1*). Nonhuman primates (NHPs) are not primary reservoirs of rabies; nevertheless, 159 reports of rabies in NHPs, of which 134 were laboratory-confirmed cases, have been retrieved from various sources in South America, Africa, and Asia (*2*). This total is probably underestimated because weak rules pertaining to rabies surveillance in some countries, such as not requiring reports of rabidity in all species, are likely to result in underreporting of rabid NHPs. Cases of rabies in humans after injury by NHPs were also reported in 25 persons, mostly in Brazil (*2*). Although rarely reported, documented cases of rabies infections in NHPs and subsequent transmission to humans do occur, warranting the need for rabies postexposure prophylaxis (PEP) after NHP exposure in countries to which rabies is endemic. The epidemiology of NHP-related injuries has been described in case reports or small cohorts of patients only (*3*,*4*). Here, we describe the epidemiology of 135 cases of NHP-related injuries in persons seen in the Marseille Rabies Treatment Centre in Marseille, France.

## The Study

During 2001–2014, epidemiologic data on NHP–related injuries and associated rabies PEP treatment were prospectively collected for patients of the Marseille Rabies Treatment Centre. Demographics, place of exposure, travel characteristics, clinical data, and rabies PEP were documented.

A total of 135 cases of persons injured by NHPs reported during a 14-year period were included, representing an average 10 cases/year (range 4–16 cases/year), with a tendency to increase over time (Figure, panel A). Exposures were more frequently reported during July–October (Figure, panel B). The F/M sex ratio of patients was 1.1, and the mean age was 30 years (median 27 years; range 3–70 years); 20.7% of patients were <15 years of age (Table 1). Of the 135 patients, 2 had complete and 1 incomplete preexposure vaccination against rabies. Most exposures (n = 120, 88.9%) occurred outside of France, notably in Southeast Asia (n = 82); most cases occurred in Thailand (n = 48) and Indonesia (n = 25). Most exposures in Thailand were on Monkey Beach on Koh Phi Phi Island and in the Lopburi Monkey Temple; most exposures in Indonesia were in the Ubud Monkey Forest in Bali (Table 2). Most persons injured abroad were tourists with a mean travel duration of 21 days (median 17 days; range 3–180 days). The mean time between the first date of travel and exposure was 10 days (median 8 days; range 1–50 days).

**Figure:**
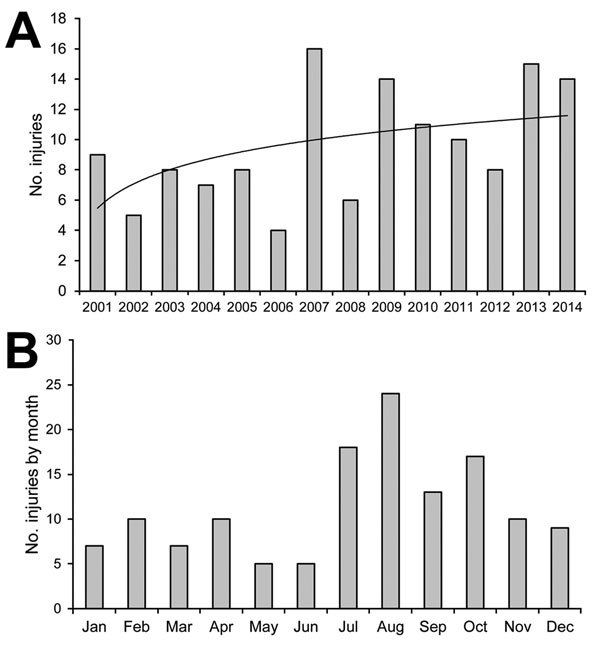
Number of injuries to humans by nonhuman primates requiring rabies postexposure prophylaxis, Marseille Rabies Treatment Centre, Marseille, France, 2001–2014. A) Logarithmic regression was used to calculate a line of best fit of y = 2,3191ln(x) + 5.4699 (black line). B) Cumulative occurrence by month.

**Table 1:** Demographic, vaccination status, place of exposure, and travel characteristics of 135 persons injured by nonhuman primates requiring rabies postexposure prophylaxis, Marseilles, France, 2001–2014

Characteristics	No. (%)
Female sex	70 (51.9)
Age group, years	
0–15	28 (20.7)
16–39	71 (52.6)
40–59	27 (20.0)
60 and over	9 (6.7)
Preexposure vaccination against rabies	2 (1.5)
Place of exposure	
France	15 (11.1)
Thailand	48 (35.7)
Indonesia	25 (18.5)
Other countries in Asia*	14 (10.4)
North Africa†	13 (9.6)
Sub-Saharan Africa‡	11 (8.1)
Central and South America and Caribbean§	6 (4.4)
Spain/Rock of Gibraltar	3 (2.2)
Reason for travel, n = 120	
Tourism	99 (82.5)
Other¶	6 (5.0)
Not documented	15 (12.5)
Days of travel duration, n = 119#	
0–7	6 (5.0)
8–14	21(17.6)
15–21	43 (36.2)
21–28	19 (16.0)
>29	14 (11.8)
Not documented	16 (13.4)
Days between first date of travel and exposure, n = 119#	
0–7	41 (34.5)
8–14	33 (27.6)
15–21	17 (14.3)
>22 d	4 (3.4)
Not documented	24 (20.2)

*Vietnam (5), India (4) Cambodia (3), Myanmar (1), Sri-Lanka (1). †Morocco (10), Algeria (3). ‡Madagascar (4), Kenya (4), Botswana (1), Cameroon (1), Central African Republic (1). §Brazil (2), Anguilla (1), Dominican Republic (1), Mexico (1), Peru (1). ¶Persons, or their descendants, who have immigrated to Marseille from another country who were exposed while visiting friends and relatives in their country of origin (3), expatriate (1), humanitarian worker (1), and student (1). #Expatriate traveler was excluded.

**Table 2:** Detailed place of exposure of 73 travelers injured by nonhuman primates in Thailand and Indonesia requiring rabies postexposure prophylaxis, Marseille, France, 2001–2014

Location	No.
Thailand, total	48
Koh Phi Phi	
Monkey Beach	15
Lopburi	
Lopburi Monkey Temple	8
Bangkok	
Park	2
Zoo	1
Temple	1
Koh Samui	
Monkey show	2
Koh Phangan	
Undocumented	2
Phuket	
Patong Beach	2
Other	
Temple, Chiang Mai	1
Street, Korat	1
Temple, Singburi	1
Temple, Sukhothai	1
Not documented	11
Indonesia, total	49
Bali	24
Ubud sacred monkey forest sanctuary	17
Temple, Uluwatu	2
Temple, undocumented	2
Undocumented	3
Not documented	1

Injuries most commonly occurred on the upper limbs (57.8%); subsequent occurrences of injury were on lower limbs (28.9%), head (5.9%), trunk (3.7%), or multiple sites (2.2%). Details of 2 cases were not documented. Most injuries (66.7%) were severe transdermal type 3 injuries according to the WHO classification, resulting from severe bites (*1*); the remaining (33.3%) were type 2 injuries (minor abrasions and scratches without bleeding). According to WHO guidelines (*1*), rabies immune globulin (RIG) was indicated for 65.2% of patients who had type 3 injuries and no previous vaccination against rabies. Among patients injured abroad, 58 (48.3%) started rabies PEP abroad; the mean time between injury and treatment was 1 day (median 0 days; range 0–21 days). Most (77.6%) underwent the Essen protocol and 17.2% underwent the Zagreb protocol (*1*); and 5.2% underwent the 7-dose mouse brain vaccine protocol (local subcutaneous and intradermal protocol used in North Africa: seven 1.0-mL subcutaneous injections from day 0 through day 6, followed by 2-site 0.1-mL intradermal injections on days 10, 14, 29, and 90). A total of 35 patients (25.9%) had an indication for RIG, of whom 8 (22.9%) received the RIG abroad. Among the 27 remaining patients, the mean time between first injection of vaccine abroad and the first consultation in France was 12 days (median 12 days; range 3–31 days). An additional 6 patients (17.1%) received RIG in France, and the remaining 21 patients (60.0%) did not. Of those 21 patients, 20 sought follow-up medical consultation in France >7 days after the first vaccine was provided abroad; the initiation of RIG is contraindicated if >7 days have passed since vaccine initiation (*5*). A total of 62 patients started their treatment in France with a mean time between injury and treatment of 15 days (median 11 days; range 2–123 days). Most underwent the Essen protocol (71.0%), followed by the Zagreb protocol (27.4%). One patient who received complete preventive vaccination received 2 booster doses at days 0 and 3. Of 44 patients for whom RIG was indicated, 39 (88.6%) received RIG. No cases of rabies were observed among the 135 persons injured by NHPs.

## Conclusions

NHP bites among travelers are not infrequent. An estimated 31% of injuries requiring rabies PEP among international travelers are caused by NHPs, which rank second after dogs in most studies and first in studies conducted among travelers returning from Southeast Asia (*2*). In a recent GeoSentinel survey involving 2,697 travelers requiring rabies PEP, NHPs accounted for 66% of animal bites occurring in Southeast Asia, and tourists made up a disproportionately large portion of the NHP exposures (92%) (*6*).

Bites by NHPs can transmit other potentially life-threatening zoonoses, such as the herpes B virus (*Cercopithecine herpesvirus* 1). The human herpes B virus infections that have been described have all occurred after contact with macaques in a biomedical research setting (*7*). However, samples of >80% of macaques at the Monkey Forest in Bali tested positive for antibodies against the herpes B virus, a naturally occurring infectious agent that is endemic among macaque monkeys from Asia (*8*). Appropriate B virus prophylaxis may be considered for travelers injured by Asian macaques, although this treatment is not applicable to those injured by New World NHPs (*3**,**4*).

In this study, we observed that most exposures occurred among adult tourists to Southeast Asia during the summer months, which probably reflects travel volume. Furthermore, we identified 2 major tourist destinations in Thailand and 1 in Indonesia as locations where monkeys were most likely to bite tourists, reflecting the regional distribution of both monkey and tourist populations. Most patients were taking relatively short trips and were bitten within the first 10 days after their arrival. Indeed, monkey bites are relatively frequent at these tourist places. An estimated 6% of visitors to Bali monkey temples are bitten by macaque monkeys (*9*). No detailed information is available about documented cases of rabies among monkeys at these tourist destinations; however, confirmed cases have been reported in monkeys as well as in gibbons and langurs in Thailand (Panichabhongse P., The epidemiology of rabies in Thailand [thesis]. 2001 [cited 2015 Jun 1] (http://mro.massey.ac.nz/bitstream/handle/10179/4545/02_whole.pdf?sequence=1&isAllowed=y). We show that most of the cases involved persons who had not received preexposure immunization and that ≈33% of patients for whom RIG was indicated did not receive RIG. Only a small proportion of travelers received RIG in the country of exposure, which reflects the limited supply of this biological agent in many countries. 

Unvaccinated Western travelers who are unaware of the risk for rabies regularly engage in contact with animals during their trips, often resulting in expensive PEP. To decrease the need for rabies PEP after animal bites, it is crucial that travelers to countries in which rabies is endemic be fully informed of this specific risk, which can be easily minimized by avoiding contact with animals, avoiding feeding them, avoiding smiling at them (showing teeth is a sign of aggression), avoiding dropping something that a monkey has grabbed, and avoiding showing fear. Our findings indicate that tourists should be given strong warnings before travel to visit Koh Phi Phi, Lopburi, or Ubud.
